# Supplementary material for: Rescue of a Rotenone Model of Parkinson’s Disease in C. elegans by the Mitochondrial Na+/Ca2+ Exchanger Inhibitor CGP37157
Source: Int J Mol Sci. 2025 Apr 4;26(7):3371. doi: 10.3390/ijms26073371 (PMC11989483; doi:10.3390/ijms26073371)
Supplement: Supplementary file 1 [file ijms-26-03371-s001.zip › ijms-3480899-supplementary.pdf]

Supplementary Table S1

	Half-life (days)	N (count/total)	% Half-life vs control	p-value vs control	% Half-life vs rotenone	p-value vs rotenone
**Control**	17,659	138/146				
**Control + CGP 25 µM**	16,977	141/155	↓ 3,9	n.s.	↑ 53,0	<0.001
**Rotenone**	11,097	171/191	↓ 37,2	<0.001		
**Rotenone + CGP 25 µM**	16,611	139/169	↓ 5,9	n.s.	↑ 49,7	<0.001
**Control**	18,261	116/133				
**Control + CGP 25 µM**	16,344	120/135	↓ 10,5	<0.005	↑ 69,7	<0.001
**Rotenone**	9,631	204/220	↓ 47,3	<0.001		
**Rotenone + CGP 25 µM**	13,406	164/199	↓ 26,6	<0.001	↑ 39,2	<0.001
**Control**	19,260	117/124				
**Control + CGP 25 µM**	18,519	11/119	↓ 3,85	n.s.	↑ 114,0	<0.001
**Rotenone**	8,652	119/127	↓ 55,1	<0.001		
**Rotenone + CGP 25 µM**	13,862	136/148	↓ 28,0	<0.001	↑ 60,2	<0.001

Table S1. Lifespan assays performed with Control, CGP37157, rotenone and rotenone + CGP37157 in wild-type worms. The table shows three separate lifespan assays performed with control worms and worms treated with CGP37157, rotenone or rotenone+CGP37157. In each assay, around 100 synchronized young adults (day 1) per condition were transferred to the plates (10 worms/plate). Control and drug-containing assays were always carried out in parallel at 20ºC in a temperature controlled incubator. Plates were scored for dead worms every day. In each assay we show the half-life of the worms in each condition obtained from the Kaplan-Meier analysis (SPSS software), the number of worms in each condition (counted/total, the difference corresponds to censored worms), the percent changes in the half-life in the drug-containing conditions, and the significance of the differences obtained from the log-rank test.

Supplementary Table S2

	**Fig. 1B**				
**F**	27.36				
**P**	0.00015				
	**Fig. 2A**	**Fig. 2C**	**Fig. 2E**	**Fig. 2F**	**Fig. 2G**
**F**	16.40	11.67	6.43	8.14	6.35
**P**	<0.0001	<0.0001	0.0003	<0.0001	0.0013
	**Fig. 3B**	**Fig. 3C**	**Fig. 3D**	**Fig. 3E**	
**F**	10.42	7.81	5.31	8.11	
**P**	0.0001	0.0011	0.0075	0.0008	
	**Fig. 4A**				
**F**	32.89				
**P**	<0.0001				
	**Fig. 5A**	**Fig. 5B**	**Fig. 5C**	**Fig. 5D**	**Fig. 5E**
**F**	10.34	11.46	11.92	1588	84.75
**P**	0.0002	<0.0001	<0.0001	<0.0001	<0.0001
	**Fig. 6A**	**Fig. 6B**	**Fig. 6C**		
**F**	25.71	40.16	39.98		
**P**	<0.0001	<0.0001	<0.0001		
	**Fig Sup 3A**	**Fig Sup 3C**			
**F**	250	259			
**P**	<0.0001	<0.0001			

Table S2. F and P values of the One-Way ANOVA tests carried out on the experimental data shown in all the Figures.

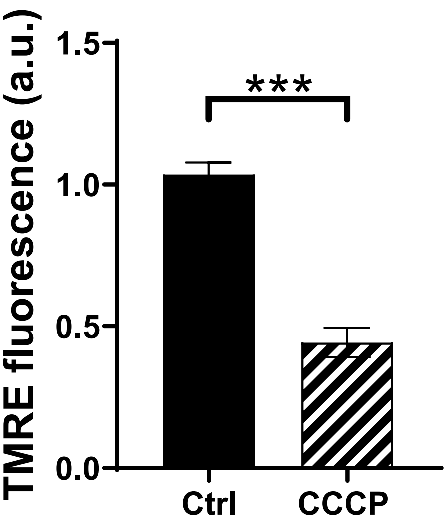

Supplementary FigureS1. Effect of complete depolarization with the protonophore CCCP on mitocondrial membrane potential measured with TMRE fluorescence. Data are means of 3 different experiments in each condition. Data are mean ± s.e.m. Significance was obtained using T-test. *****, p<0,005.**

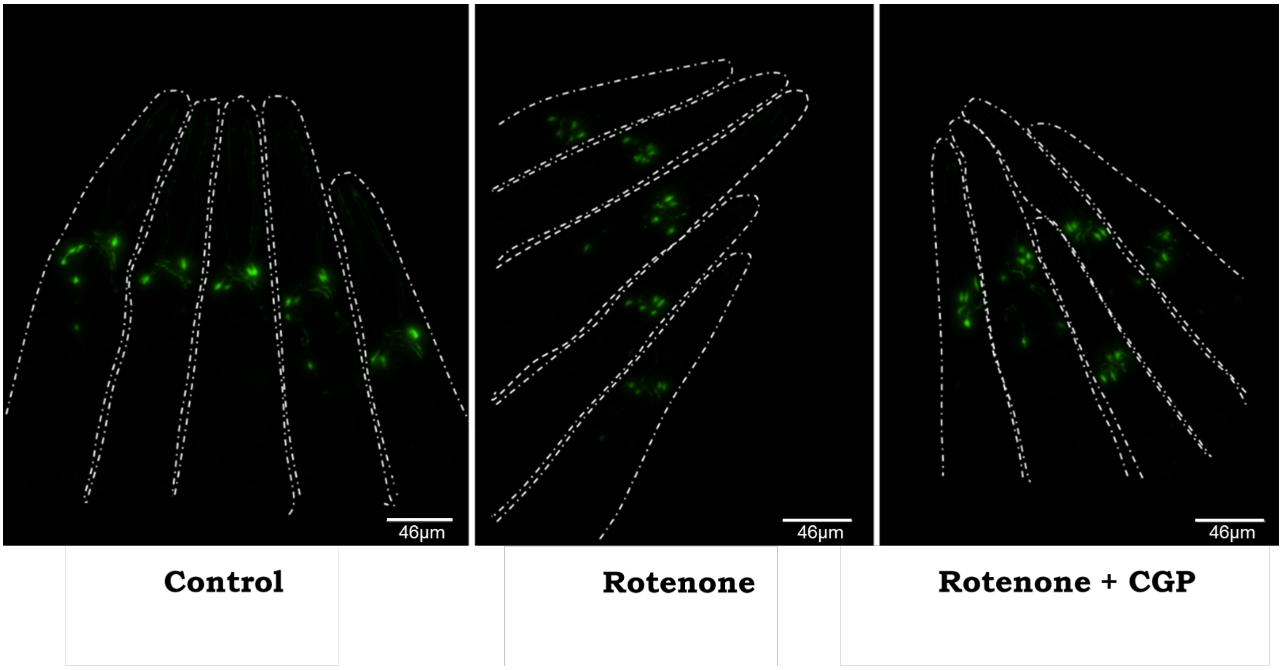

Supplementary Figure S2. Typical image showing the changes in the fluorescence of the dopaminergic neurons of the BZ555 strain induced by rotenone or rotenone + CGP37157. Z-stacks of CEP and ADE dopaminergic neurons were acquired using an epifluorescence microscopy ECLIPSE Ni-E (Focusing Nosepiece System) with an attached NIKON DS-Ri2 camera, 20X objective, and a FITC filter cube (475nm/509nm excitation/emission wavelengths). Around 20 Z-stack images of 1 μm were projected using the Image J tool 'Z-projection' to generate complete dopaminergic neuron images.

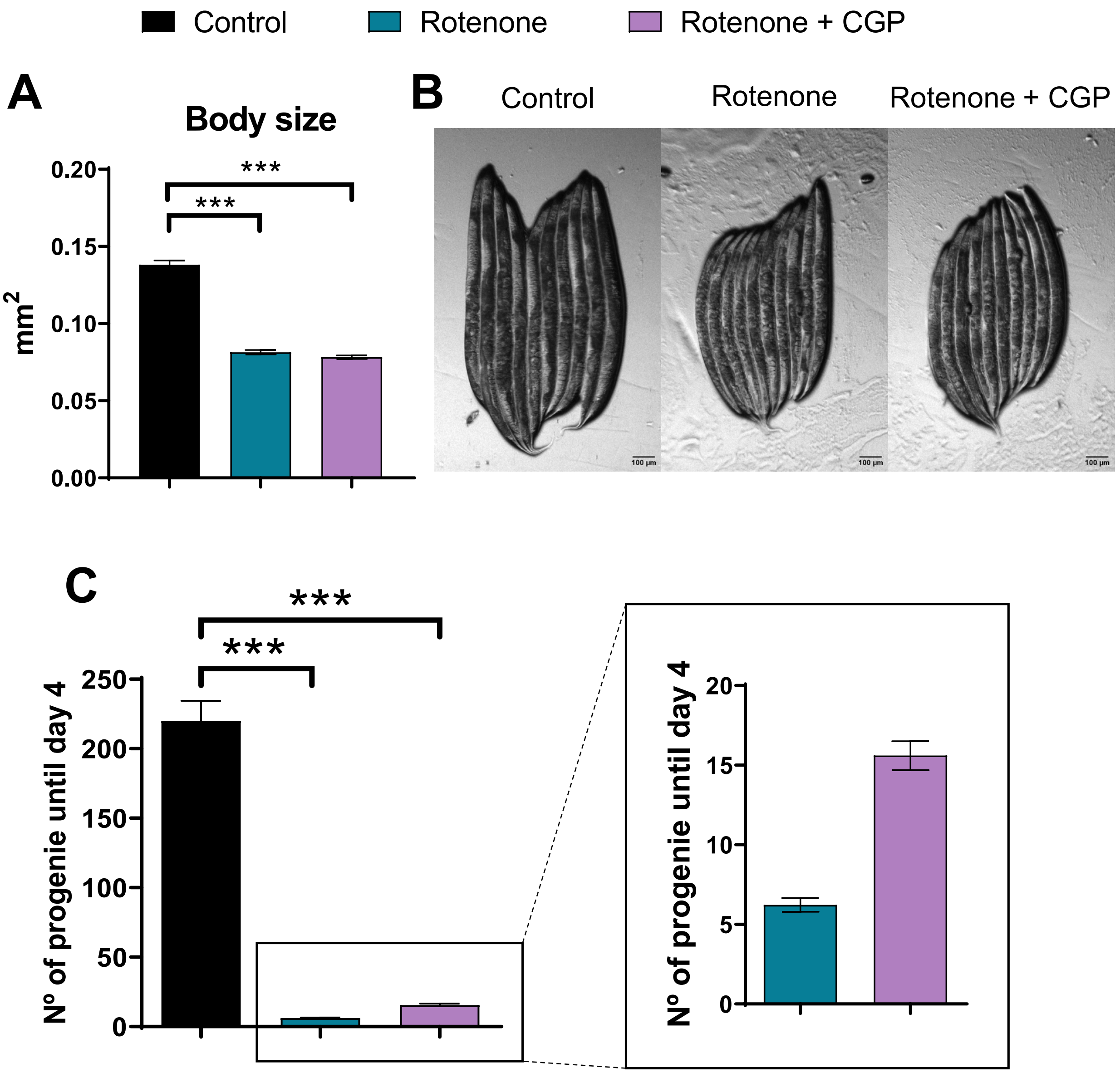

Supplementary Figure S3. Effect of rotenone and CGP37157 on *C. elegans* size and fertility. Fig. S1A shows the decrease in body area of day 5 worms induced by rotenone, which was not modified by CGP37157. Body size was measured using the ImageJ plugin "wrMTrck" also used to monitor mobility. Data are mean±sem (n=55-198). Fig. S1B shows images of the worms in the three different conditions. Fig. S1C shows the number of eggs produced by worm in the three conditions. Fertility was measured in N2 worms grown without FUdR and in the presence or in the absence of rotenone and/or CGP37157. They were placed 1 worm/well in 24-well plates, and then changed to new wells every day, during 4 days. After that, eggs and larvae in each well were counted. Data are mean±sem (n=17-22). Significance was obtained using ANOVA test and post hoc comparisons made with Tukey's test. ***, p<0,005.
